# Which Physiological Swallowing Parameters Change with Healthy Aging?

**DOI:** 10.21926/obm.geriatr.2101153

**Published:** 2021-01-19

**Authors:** Renata Mancopes, Pooja Gandhi, Sana Smaoui, Catriona M. Steele

**Affiliations:** 1. Swallowing Rehabilitation Research Laboratory, KITE Research Institute — Toronto Rehabilitation Institute — University Health Network, 550 University Avenue, 12^th^ floor, Toronto, Ontario, Canada, M5G 2A2; 2. Rehabilitation Sciences Institute, Temerty Faculty of Medicine, University of Toronto,500 University Avenue, Suite 160, Toronto, ON, Canada, M5G 1V7

**Keywords:** Deglutition, swallowing, aging, videofluoroscopy, kinematics, presbyphagia

## Abstract

Research suggests there are age-related changes in swallowing that do not constitute impairment (“presbyphagia”). The goal of this study was to explore the influence of age on quantitative measures of healthy swallowing by controlling for the effects of sex and sip volume in order to determine the specific characteristics of presbyphagia. Videofluoroscopy recordings of thin liquid swallows from 76 healthy adults (38 male), aged 21–82 were analysed. Blinded duplicate ratings of swallowing safety, efficiency, kinematics, and timing were made using the ASPEKT method. Hierarchical regression models were used to determine the effects of age, sex, and sip-volume on swallowing. There were no age-related changes in sip volume, number of swallows per bolus, frequency or severity of penetration-aspiration, duration of the hyoid-burst (HYB)-to-upper-esophageal-sphincter (UES) opening interval, time-to-laryngeal-vestibule-closure (LVC), peak hyoid position, hyoid speed, or pharyngeal residue. Significant changes seen with increasing age included: longer swallow reaction time, UES opening duration and LVC duration; larger pharyngeal area at rest and maximum constriction; and wider UES diameter. Male participants had larger sip volume and pharyngeal area at rest. Larger sip volumes were associated with multiple swallows per bolus and shorter hyoid-burst-to-UES opening intervals. These results help to define presbyphagic changes in swallowing that can be expected in healthy older adults up to 80 years of age, and distinguish them from changes that represent impairment. Certain parameters showed changes that were opposite in direction to changes that are usually considered to reflect impairment: longer UES opening, longer LVC duration and wider UES opening. These changes may reflect possible compensations for slower bolus transit. Further research is needed to determine the points along the age continuum where observed age-related changes in swallowing begin to emerge.

## 1. Introduction

It is estimated that between 1970 and 2025 there will have been an increase of 223% in the elderly population worldwide, around 694 million people [1]. Despite the major medical advances that have made this possible, it is a great social and economic challenge to keep the older population healthy. We know that aging is a natural process, and that it includes many physical and psychological changes that impact health, survival and quality of life. Changes also occur in sensory and motor functions involved in swallowing, with potential impact on nutrition, pulmonary health and participation in social functions involving eating and drinking. In the literature, the term presbyphagia has been used to refer to changes in swallowing that are part of the natural process of aging, and reflect age-related degeneration in nerves and muscles [2]. Although such changes are not, in and of themselves, pathologic, it has been argued that they constitute a vulnerability or reduction in physiologic reserve that places seniors at increased risk for functional swallowing impairments, with associated risks of morbidity and mortality [3–6]. If we consider the projected growth of the world’s elderly population, it is clear that the number of older individuals who are likely to present with swallowing difficulties will grow significantly. Consequently, it becomes important to delineate the kinds of changes that are expected as a function of age in healthy adults, in order to differentiate presbyphagia from changes that reflect concerns requiring clinical assessment and intervention [7].

Oropharyngeal dysphagia is characterized by two primary functional impairments: a) impaired swallowing safety, involving penetration or aspiration of material into the larynx and lower respiratory tract; and b) impaired swallowing efficiency, where poor bolus clearance leads to pharyngeal residue [8, 9]. Although penetration-aspiration and residue are usually interpreted to represent impairment, several studies suggest that these phenomena may be seen in a small percentage of healthy adults on videofluoroscopic or endoscopic examination, with higher frequency in healthy older adults [10–13]. In order to better understand these apparent changes in swallowing function, it is also important to determine whether there are age-related changes in the underlying anatomy and physiology of swallowing.

Older adults are known to experience a loss of muscle bulk and strength in the limb musculature (sarcopenia) [14]. A similar phenomenon has been observed in the tongue and pharyngeal musculature with a loss of muscle bulk [15], decreasing pharyngeal wall thickness [16] and corresponding increases in pharyngeal lumen volume [16]. Associated age-related changes in function include reductions in maximum isometric tongue pressure generation capacity [15, 17, 18], and reductions in pharyngeal constriction [16, 19], which have been associated with increased pharyngeal residue [19]. Interestingly, however, current evidence suggests that older age does not appear to be associated with reduced amplitudes of pharyngeal lumen pressure during bolus transit, but rather the opposite, introducing the possibility that some changes seen with age may not be towards the direction of impairment, but rather, may reflect compensation [20].

There is consensus in the literature that changes in structural movements during swallowing, as seen on videofluoroscopy, include reduced diameters of upper esophageal sphincter (UES) opening [21]. However, there are mixed reports regarding both the presence and direction of changes in the extent of hyoid and laryngeal movement [22–25]. With respect to timing measures, a recent review summarized reports of age-related changes across 32 different measures [26], showing a large degree of variability; differences in methodology and in operational definitions across studies were noted to limit the opportunity for data pooling and meta-analysis. Notwithstanding these limitations, the authors concluded that the effects of aging on swallow timing were limited to: a) significantly delayed onset of the pharyngeal swallow leading to longer measures of swallow reaction time (the interval between bolus arrival in the pharynx and the onset of hyoid excursion associated with a swallow); and b) longer durations of UES opening.

The most common approach to examining the effects of aging on swallowing has been to compare data across cohorts. The age-ranges for these cohorts have been largely arbitrary and not contiguous, guided by social definitions of advanced age (such as retirement age) and convenience sampling strategies. Some studies suggest that there may be more extreme changes in the “very old”, meaning individuals above the age of 85 [13, 27]. Very few studies have examined age as a continuous construct, exploring the full range for evidence of trends that are best explained by advancing age. In our laboratory, we use a standard videofluoroscopy protocol and analysis method known as the ASPEKT Method (Analysis of Swallowing Physiology: Events, Kinematics and Timing) [28] to characterize oropharyngeal swallowing physiology across the range from thin to extremely thick liquids. We have recently published reference data for a comprehensive set of 17 parameters in healthy adults under the age of 60 years [29, 30]. In this article, we extend that work to explore thin liquid swallowing across a broader age-range, incorporating data from healthy adults aged 60–82.

Our goal was to identify parameters that display systematic changes across the adult age continuum, thereby pointing to changes that should be expected in presbyphagia. Furthermore, we were interested to explore changes both in the direction of impairment (or functional limitation) and changes in the opposite direction, which might reflect spontaneous compensations that help to preserve function in healthy aging. Based on previous literature, we hypothesized that we would see the following changes with increasing age: increased frequency of penetration; longer swallow reaction times; longer durations of laryngeal vestibule closure (LVC) and UES opening duration; reduced pharyngeal constriction (i.e. larger pharyngeal area at the point of maximum pharyngeal constriction); larger pharyngeal area at rest; reduced diameter of UES opening; increased frequency of a pattern of multiple swallows (i.e. > 1) per bolus; and increased pharyngeal residue. We did not expect to see age-related changes in sip volume, in other measures of swallow timing (time-to-LVC, the hyoid-burst-to-UES-opening interval) or in measures of hyoid kinematics.

## 2. Materials and Methods

### 2.1 Participants

Data were collected from a sex-balanced sample of 38 healthy adults aged 60-plus (range 61–82) and pooled with the under-60 reference data (n=38) previously described by Steele and colleagues [28]. Individuals who reported any history of motor speech, gastroesophageal or neurological impairment, radiation or surgery to the head and neck, swallowing impairment, chronic sinusitis, altered taste, insulin-dependent diabetes, current pregnancy or known allergies to ingredients in the study stimuli were excluded. The study protocol received human subjects approval from the local institutional ethics review board (UHN CAPCR ID 15–9431).

### 2.2 Data Collection

Each participant underwent a standard videofluoroscopy examination beginning with 3 discrete boluses of 20% w/v thin liquid barium prepared with Bracco Diagnostics E-Z-PAQUE powdered barium sulfate (96% w/w) and Nestlé Pure Life bottled water. For each bolus, the instruction was to take a comfortable sip and swallow when ready, without waiting for a cue. Sip volume was calculated by comparing measures of cup weight taken before and after each bolus on a digital balance. The videofluoroscopy recordings were acquired using 30 pulses per second and recorded at 30 frames per second. Separate recordings were made for each bolus. The final dataset contained 226 thin boluses. Issues with recording quality resulted in missing data for 1 thin bolus each from a 28 year-old female and a 53 year-old male in the under-60 cohort.

### 2.3 Videofluoroscopy Rating

The recording for each bolus was saved as a separate file and randomly assigned to two trained speech-language pathologists for rating. Raters were blinded to participant, bolus order and each other’s ratings. Rating involved 3 stages. In the first stage, raters identified the number of swallows for each bolus, the worst Penetration-Aspiration Scale [31] score across the observed swallows for each bolus, and the frame numbers corresponding to a series of key events in the swallow sequence. Strict thresholds for inter-rater agreement were applied to each measure as described previously [28], and consensus meetings were convened to resolve any discrepancies. In the second stage, the confirmed frame numbers for maximum UES opening, maximum pharyngeal constriction, and the swallow rest frame marking the end of each swallow were extracted for pixel-based measurement of UES diameter, pharyngeal area at maximum constriction, pharyngeal area at rest and pharyngeal residue on the frame of swallow rest. Measurement discrepancies exceeding pre-specified thresholds were again flagged and resolved by consensus. The third stage involved frame-by-frame tracking of hyoid position, beginning 10 frames prior to hyoid burst onset until 10 frames after the “swallow rest” frame marking the end of each swallow. All pixel-based measures were scaled to the length of the individual participant’s C2–4 cervical spine [32]. The resulting dataset comprised 19 parameters per bolus, as listed in Table 1.

### 2.4 Analysis

#### 2.4.1 Inter-Rater Agreement

Inter-rater reliability for Penetration-Aspiration Scale scores, event identification (frame numbers) and pixel-based line and area measures was calculated based on ratings obtained prior to discrepancy resolution using measures of % absolute agreement, mean absolute difference and intra-class correlations.

#### 2.4.2 Statistical Analysis

For all parameters, we computed descriptive statistics for the 60-plus cohort (frequencies for categorical parameters; medians and interquartile range for continuous parameters) to enable comparison with previously-published descriptive statistics for the under-60 cohort [29]. We plotted histograms across the full age range (i.e. 21–82) to understand the distribution of scores and to see whether there was sufficient spread to run regression analyses, either logistic (for categorical parameters) or linear (for continuous parameters). Additionally, we performed Pearson correlations between all pairwise combinations of continuous dependent variables to determine whether corrections to the alpha-level would be needed to adjust for non-independence in the planned regression models. We applied Bonferroni corrections to the *p*-values wherever correlations of r > 0.25 were observed, by dividing the 0.05 by the number of related parameters. We calculated participant mean values for each parameter for use in the regression models, which we conducted following a hierarchical approach. We examined the influence of age as a continuous predictor first. We then added predictors of sex and sip volume to the model in a stepwise fashion, to understand the influence of these additional predictors, and whether they accounted independently for a significant portion of the observed variance.

## 3. Results

### 3.1 Inter-Rater Agreement

For ratings of swallowing safety, absolute pre-consensus agreement on Penetration-Aspiration Scale scores and LVC integrity was seen in 94% and 97% of ratings, respectively. For event identification, pre-consensus differences in frame selection across raters were within 3 frames (on average) for the majority of events, with intra-class correlation coefficients (ICCs) of 0.98 or higher. Three events showed poorer agreement for frame identification prior to discrepancy review, with a mean absolute difference of 5 frames: LVC offset; maximum UES opening; and the frame of swallow rest. Pre-consensus absolute agreement for pixel-based measures of UES diameter were within 4%(C2–4) with an ICCs of 0.96 in 78% of cases. Pixel-based measures of pharyngeal area showed good agreement across raters, with mean pre-consensus absolute agreement within 1%(C2–4)^2^ at maximum constriction (ICC = 0.83) and within 10%(C2–4)^2^ at rest (ICC = 0.85). Pre-consensus absolute agreement for pixel-based measures of residue was within 1%(C2–4)^2^ in 85% of cases (ICC = 0.94).

### 3.2 Descriptive Statistics

Descriptive statistics for all parameters in this study have been previously published for the younger cohort [28–30]. Table 2 shows corresponding median and interquartile range boundary scores for the 60-plus cohort.

### 3.3 Correlations between Continuous Parameters

Table 3 lists the parameters where correlations larger than r = 0.25 were observed, suggesting a need to adjust p-values for the subsequent regression models to correct for non-independence. Based on the relationships that were found, Bonferroni-corrected p-values for the regression were set at *p* < 0.017 (i.e. 0.05/3) for the analyses of Hyoid-burst-to-UES-Opening-Interval, Hyoid burst duration and Time-to-LVC; *p* < 0.01 (i.e. 0.05/5) for the analyses of UES-Opening-Duration, LVC Duration, UES Diameter, Peak XY Hyoid Position and Hyoid Speed; and *p* < 0.01 (i.e. 0.05/5) for the analyses of pharyngeal area at rest and maximum constriction, and for measures of pharyngeal residue.

### 3.4 Regression Analyses

Table 4 illustrates the overall pattern of results from the hierarchical regression models.

#### 3.4.1 Sip Volume

Linear regression showed a statistically significant sex difference in sip volume (with larger sips in male participants) but no significant differences as a function of age. On average, male participants took sips that were 4.69 ml larger than women (*p* < .005).

#### 3.4.2 Number of Swallows per Bolus

Binary logistic regression was used to determine the influence of age, sex and sip volume on the number of swallows per bolus (single or multiple). There were no significant effects of age or sex. Sip volume had a significant effect (*p* < 0.001), with each additional ml of increasing the odds of multiple swallows by a factor of 0.09. Post-hoc inspection of the data showed that the odds of multiple swallows per bolus were 2.8-fold higher for sips larger (versus smaller) than 16 ml (95% confidence interval: 1.4 to 5.7).

#### 3.4.3 Penetration-Aspiration and Integrity of Laryngeal Vestibule Closure

Penetration was extremely rare in the sample, with 89% of the participants showing maximum PAS scores of 1 or 2 across all thin liquid swallows. There were no occurrences of aspiration in the sample. Similarly, laryngeal vestibule closure was determined to be complete on 97% of the boluses in the sample, with the remaining 3% classified as partially closed. Thus, there were insufficient cases of penetration or of impaired LVC to warrant logistic regression analyses to explore the influences of age, sex or sip volume.

#### 3.4.4 Timing Measures

Age was found to have a statistically significant effect on swallow reaction time (*p* < 0.05), UES Opening duration (*p* < 0.005) and LVC duration (*p* = 0.01), accounting for 6%, 12% and 8% of the observed variance, respectively. Each additional year of age contributed to a 4 ms lengthening of swallow reaction time, a 1.4 ms lengthening of UES Opening Duration and a 2.4 ms lengthening of LVC duration. The addition of sex and sip volume to the regression models showed no independent effects on these parameters and did not improve model prediction significantly. No significant effects of age were observed on the Hyoid-Burst-to-UES-Opening interval, or Time-to-LVC. Sex and sip volume showed no significant effects on Time-to-LVC but sip volume accounted for 13% of the variance in the Hyoid-Burst-to-UES-Opening interval, with a 2.4 ms reduction in this interval for each ml increase in sip volume (*p* < 0.001).

#### 3.4.5 Hyoid Kinematics

None of the regression models accounted significantly for variation in peak XY hyoid position or hyoid XY speed.

#### 3.4.6 Pixel-based Measures of UES Opening Diameter, Pharyngeal Area and Residue

##### UES Diameter.

Age was found to account for 10% of the observed variance in UES diameter (*p* < 0.005). Sex did not influence this parameter, but sip volume accounted for an additional 6% of the observed variance (*p* = 0.01). For each added year of age, UES diameter increased by 0.11%(C2–4). For each added ml of sip volume, UES diameter increased by 0.27%(C2–4).

##### Pharyngeal Area.

Age explained 10% of the observed variance in pharyngeal area at maximum constriction (*p* < 0.005) and 10% of the observed variance in pharyngeal area at rest (*p* < 0.01). With each additional year of age, measures of unobliterated pharyngeal area at maximum constriction increased by 3.5%(C2–4)^2^, while measures of pharyngeal area at rest increased by 0.31%(C2–4)^2^. There were no significant effects of sex or sip volume on measures of pharyngeal constriction, however sex accounted for an additional 7% of the observed variance (*p* = 0.01) in pharyngeal area at rest, with the measures in male participants, being 11%(C2–4)^2^ larger, on average. Sip volume was not explored for the pharyngeal area at rest measure, which is taken in a rest position, independent of swallowing activity.

##### Pharyngeal Residue.

The regression models failed to identify any significant influence of age, sex or sip volume on total pharyngeal residue. Given the lack of significant results in the composite measure, regression models for the component measures of vallecular, pyriform or other pharyngeal residue were not performed.

## 4. Discussion

The results of this analysis confirm several of our hypotheses. Specifically, the data showed longer swallow reaction times, LVC duration and UES opening duration with increasing age. Predictions of larger pharyngeal area at maximum constriction and at rest were also confirmed. Contrary to predictions, we saw larger rather than reduced diameters of UES opening as a function of advancing age. Equally interesting, however was the absence of age-related changes in other parameters. Here, predictions of more frequent penetration, multiple swallows per bolus and pharyngeal residue were not confirmed in this sample of healthy adults on thin liquid swallows. As predicted, no influence of age was seen on sip volume, time-to-LVC, the hyoid-burst-to-UES-opening interval, or hyoid kinematics. The analysis did, however, reveal independent effects of sex on sip volume and pharyngeal area at rest, and of sip volume on the hyoid-burst-to-UES-opening interval and UES diameter.

These findings are largely consistent with those reported in previous studies, but a few differences are worth discussion. First, the finding of larger rather than reduced UES diameter is consistent with findings reported by Leonard et al. [33] but appears contrary to results from a manofluorographic study by Kern and colleagues [21], who reported reduced UES opening in the antero-posterior direction, with corresponding reductions in anterior hyoid and laryngeal excursion in 14 healthy older adults aged 70–90, compared to a cohort of younger controls. A number of differences in study methodology may account for the differences in results, including use of controlled 5- and 10-ml boluses of a thickened liquid barium (300 centipoise), the presence of a solid state manometry catheter in the pharynx, the use of millimetre rather than anatomically scaled units of measurement, and the analysis of cohort differences rather than exploration of age as a continuous predictor.

**Figure 1:**
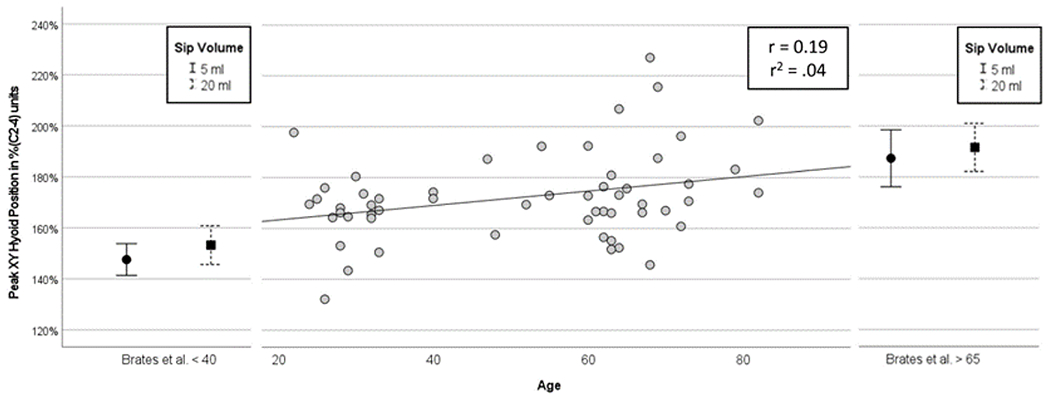
Comparison of Peak XY Hyoid Position Data between Brates et al. [25] and this study.

Second, the absence of any age-related differences in measures of peak XY hyoid position is again consistent with findings reported by Leonard et al. [33], but differs from the conclusions of a recent paper by Brates and colleagues [25], who reported larger measures of peak hyoid position using %(C2–4) units in healthy adults over age 65, compared to a control group of adults under age 40. Here again, the use of a cohort comparison analysis rather than a regression model with age as a continuous predictor may be partly responsible for the discrepancy in results. Sip volume may also be a factor, given that the Brates study involved controlled volumes of 5- and 20 ml, falling on either side of the natural sip volume range seen in our data. Figure 1 provides an illustrative comparison of peak hyoid position values across the two studies, with the mean and 95% confidence interval values for the Brates et al. [25] dataset, plotted by cohort, and a scatter plot of our data, plotted by age. The figure shows that the values from our participants fall in between the values seen for the two cohorts in the Brates et al. study, and also suggests that greater variability was present across the participants in our study, thereby diluting the apparent influence of age as a predictor.

Third, although the data revealed age-related increases in pharyngeal area at maximum constriction, this study did not find evidence of age-related increases in pharyngeal residue. This may at first appear inconsistent with previous literature in which poor pharyngeal constriction has been identified as a primary mechanism contributing to residue in patients with dysphagia [34, 35]. Although increased pharyngeal residue did not emerge as a feature of swallows in older participants, there was a significant correlation between pharyngeal constriction and pharyngeal residue, as shown in Table 3. It is also important to remember that the participants in our study were healthy, and that the analysis was limited to thin liquid boluses, which may be less likely to leave residue than thicker consistencies [28, 36].

As for any study, several limitations must be acknowledged. Perhaps most importantly, we recognize that the upper boundary of the participant age range was 82, meaning that this study does not include data for the very elderly. Additionally, because we used a convenience sample, we did not have balanced representation of participants for each decade across the range studied. Thus, although age was modelled as a continuous predictor, there were relatively fewer participants aged 40–60 than those aged outside that range. Future studies should endeavour to sample purposively from each decade and to include individuals aged 80–100, in order to reveal either incremental changes with age, or changes emerging at key points along the age continuum. Our participants were also nominally healthy, as defined by the inclusion and exclusion criteria of the study, which aimed primarily to exclude individuals medical conditions in which dysphagia is common. We cannot rule out the presence of other medical conditions in this sample [37]; as such the data should be considered representative of individuals whose health status means that they report an absence of dysphagia symptoms and dysphagia-related conditions. The results of the study are also limited to observations on naturally sized sips of thin liquid and may not be generalizable to situations where different consistencies, bolus volumes and analysis methods are used.

Notwithstanding these limitations, the data do reveal evidence of some age-related changes in swallowing that appear to be opposite in direction to changes typically seen in people with dysphagia. In particular, the findings of increased duration and diameter of UES opening, and increased LVC duration appear to suggest possible spontaneous compensations in senescent swallowing that may accommodate slower bolus transit. Certainly, these trends can be used to bolster clinical confidence that assessment findings of limited duration/diameter of UES opening and of short LVC on thin liquid swallows should not be expected in healthy older adults, and require intervention. Similarly, clinical observations of penetration-aspiration, multiple swallows per bolus, prolonged time-to-LVC, a prolonged hyoid-burst-to-UES-opening interval, reduced peak XY hyoid position or hyoid speed, and pharyngeal residue are not typical on thin liquid swallows in older adults and warrant further investigation. Conversely, some degree of prolongation in swallow reaction time measures, and of increased pharyngeal area, both at maximum constriction and at rest, is typical in older adults; clinicians need to be aware of these trends and should scrutinize these phenomena in older patients to discern situations where the values exceed the ranges seen in the current analysis.

## 5. Conclusions

In summary, this study shows that there are relatively few age-related changes in the physiology of thin liquid swallowing in healthy adults. Furthermore, with the exception of prolonged swallow reaction times and larger pharyngeal area measures at rest and during maximum constriction, the observed changes are opposite in direction to changes seen in people with dysphagia. This counters the idea that age-related changes reflect the beginning of an inevitable decline or deterioration in swallowing function. Rather, these observations appear to reveal spontaneous compensations that are at play in healthy older swallowing, and may well serve to preserve optimal swallowing function. These data help to define presbyphagia as a phenomenon that is distinct from the pathological changes in swallowing seen in individuals with dysphagia.

## Figures and Tables

**Table 1:** Videofluoroscopic parameters of swallozhewing collected in the study.

Parameter	Details
Sip volume Number of swallows per bolus	
Penetration-Aspiration Scale Score [32]	Initial swallow Maximum across all swallows for each bolus
Integrity of LVC	Complete; partial; incomplete
Swallow Reaction Time	Interval between bolus passing mandibular ramus until hyoid burst onset
Hyoid-burst-to-UES-opening interval	Interval from hyoid burst onset until UES opening
UES Opening duration	Interval from UES opening until UES closure
Time-to-LVC	Interval from hyoid burst onset until the first frame of most-complete LVC
LVC duration	Interval from the first frame of most-complete LVC until LVC offset
Peak XY hyoid position^a^	Hyoid position at maximum excursion, measured along the XY-axis, relative to the anterior inferior corner of C4, with the Y-axis defined by the C2-C4 cervical spine
Hyoid XY speed	Change from minimum to peak hyoid position along the XY axis divided bythe duration of the hyoid burst
UES diameter^a^ Pharyngeal area at maximum constriction^b^ Pharyngeal area at rest^b^	
Pharyngeal residue^b^	Valleculae Pyriform Sinuses Elsewhere in the pharynx Total pharyngeal residue (summed across component locations)

LVC = Laryngeal Vestibule Closure; UES = Upper Esophageal Sphincter.

a These distance or line parameters were calculated in anatomically scaled units, relative to the length of the C2-C4 vertebral spine, i.e. %(C2-4).

b These area measures were calculated in anatomically scaled units, relative to the squared length of the C2-C4 vertebral spine, i.e. %(C2-4)^2^.

**Table 2:** Descriptive statistics for measures of thin liquid swallowing in healthy adults aged 60-plus.

Parameter	Unit	25th %ile	Median	75th %ile
Sip volume	millilitres (ml)	9.64 ml	13.58 ml	18.31 ml
Number of swallows per bolus	number	1	1	1
Penetration-Aspiration Scale (Initial swallow)	score (1-8)	1	1	1
Penetration-Aspiration Scale (Maximum score per bolus)	score (1-8)	1	1	1
Integrity of LVC	categorical	complete	complete	complete
Swallow Reaction Time	milliseconds (ms)	100 ms	200 ms	367 ms
Hyoid-burst-to-UES-opening interval	milliseconds (ms)	67 ms	100 ms	134 ms
UES Opening duration	milliseconds (ms)	467 ms	500 ms	534 ms
Time-to-LVC	milliseconds (ms)	67 ms	134 ms	200 ms
LVC duration	milliseconds (ms)	434 ms	534 ms	634 ms
Peak XY hyoid position	%(C2-4)	161%	176%	189%
Hyoid XY speed	%(C2-4)per second	99%	117%	154%
UES diameter	%(C2-4)	17%	23%	27%
Pharyngeal area at maximum constriction	%(C2-4)^2^	0%	2%	3%
Pharyngeal area at rest	%(C2-4)^2^	51%	63%	76%
Vallecular residue	%(C2-4)^2^	0%	0%	1%
Pyriform Sinus residue	%(C2-4)^2^	0%	0%	0%
Other pharyngeal residue	%(C2-4)^2^	0%	0%	0%
Total pharyngeal residue	%(C2-4)^2^	0%	1%	2%

LVC = Laryngeal Vestibule Closure; UES = Upper Esophageal Sphincter.

**Table 3:** Parameters with correlations greater than r = 0.25.

Parameter	Correlated Parameters	r	*p*-value
Time-to-LVC	Hyoid-Burst-to-UES-opening interval	0.496	< 0.001
	LVC Duration	− 0.599	< 0.001
LVC Duration	UES Opening Duration	0.405	< 0.001
	UES Diameter	0.29	< 0.001
UES Opening Duration	UES Diameter	0.311	< 0.001
	Peak XY hyoid position	0.265	< 0.001
Peak XY hyoid position	UES Diameter	0.549	< 0.001
	Hyoid XY Speed	0.283	< 0.001
	Pharyngeal Area at Rest	0.268	< 0.001
Pharyngeal Area at Maximum Constriction	Vallecular Residue^a^	0.492	< 0.001
	Pyriform Sinus Residue^a^	0.432	< 0.001
	Total Pharyngeal Residue^a^	0.553	< 0.001

LVC = Laryngeal Vestibule Closure; UES = Upper Esophageal Sphincter

a Measures of residue in all locations, including elsewhere in the pharynx, were also significantly correlated with each other, with r > 0.3.

**Table 4:** Summary of the Regression Analyses.

Parameter	Age effect?	Sex effect?	Sip volume effect?
Sip volume	No	Yes	N/A
Penetration-Aspiration	N/A: Insufficient examples of problematic PAS Scores		
LVC Integrity	N/A: Insufficient examples of problematic LVC Integrity		
Number of swallows per bolus	No	No	Yes
Swallow Reaction Time	Yes	No	No
Hyoid-Burst-Onset-to-UES-Opening	No	No	Yes
UES Opening Duration	Yes	No	No
Time-to-LVC	No	No	No
LVC Duration	Yes	No	No
Hyoid Peak XY Position	No	No	No
Hyoid XY Speed	No	No	No
UES Maximum Diameter	Yes	No	Yes
Pharyngeal Area at Maximum Constriction	Yes	No	No
Pharyngeal Area at Rest	Yes	Yes	N/A
Total Residue	No	No	No

LVC = Laryngeal Vestibule Closure; UES = Upper Esophageal Sphincter; N/A = Not Applicable
